# Characterization of a Novel Ourmia-Like Mycovirus Infecting *Magnaporthe oryzae* and Implications for Viral Diversity and Evolution

**DOI:** 10.3390/v11030223

**Published:** 2019-03-05

**Authors:** Chang Xin Li, Jun Zi Zhu, Bi Da Gao, Hong Jian Zhu, Qian Zhou, Jie Zhong

**Affiliations:** Hunan Provincial Key Laboratory for Biology and Control of Plant Diseases and Insect Pests, Hunan Agricultural University, Nongda Road 1, Furong District, Changsha 410128, China; changxinli1998@sina.com (C.X.L.); dlxy0808@sina.com (J.Z.Z.); bdgao@aliyun.com (B.D.G.); zjz0808@gmail.com (H.J.Z.)

**Keywords:** mycovirus, *Magnaporthe oryzae*, ourmiavirus

## Abstract

Here, the molecular characterization of a novel mycovirus that was isolated from a phytopathogenic fungus *Magnaporthe oryzae* and designed as *Magnaporthe oryzae* ourmia-like virus 4 (MOLV4) is reported. MOLV4 has a genome that is 2497 bp long and possesses a single open reading frame (ORF), which encodes the product RNA-dependent RNA polymerase (RdRp). Sequence similarities were found between the MOLV4 encoded RdRp and the counterparts of a few previously reported ourmia-like mycoviruses. Virus-curing and biological comparison indicate that the virus has no or mild effects on the morphology and mycelium growth rate of the host fungus. Phylogenetic analysis using the RdRp aa sequences was performed. The results show that MOLV4 is clustered with the ourmia-like mycoviruses, forming a clade closely related to ourmiaviruses but distinct from narnaviruses. In addition, database searches revealed that several MOLV4-related sequences are present in the transcriptome shotgun assembly (TSA) library, expressed sequence tag database (ESTdb), whole-genome shotgun (WGS) library, and genomic survey sequences (GSS) libraries of a few other species of eukaryote organisms. Our results show that MOLV4, together with other similar ourmia-like mycoviruses, might represent a virus clade that links the plant ourmiaviruses and fungal narnaviruses and has a wide range of hosts.

## 1. Introduction

Mycoviruses (fungal viruses) are ubiquitous and can infect and replicate in all major taxa of fungi, including plant-pathogenic fungi [1]. Although the vast majority of mycoviruses are associated with latent infections, the infections of some mycoviruses can cause clear phenotype alterations in growth, sporulation, pigmentation, and virulence, that often lead to hypovirulence and debilitation. Moreover, a number of mycoviruses are reported to have beneficial effects on the host fungi, such as the increase of virulence (hypervirulence) in plant pathogenic fungi or oomycetes [2,3], the enhancement of competitive ability via producing killer proteins in some yeasts [4,5], and the improvement of heat tolerance in plants conferred by the symbiotic host fungus [6]. As has been shown by the successful use of Cryphonectria hypovirus 1 (CHV1) to control chest blight disease in Europe [7,8,9], mycovirus-mediated hypovirulence is considered to be potentially useful for the control of fungal diseases. During the last few decades, many researchers have been inspired to hunt mycoviruses for useful virocontrol agents. Thus, many mycoviruses were subsequently found, largely expanding the knowledge of mycoviral diversity, ecology, and evolution.

The genomes of most mycoviruses are double-stranded (ds) RNA or positive single-stranded ((+)ss) RNA [7], and several consist of DNA [10] or negative-sense RNA (ssRNA) [11]. The (+)ssRNA mycoviruses are classified into six families: Alphaflexiviridae, Barnaviridae, Gammaflexiviridae, Hypoviridae, Narnaviridae, and Mymonaviridae. With the discovery of more novel mycoviruses that cannot not be assigned to any of the established families or genera, the taxonomy of mycoviruses is regularly being refined.

Up until now, viruses in the Narnaviridae family were the simplest viruses that infected filamentous fungi, yeasts, and oomycetes [12]. Viruses in this family have a (+)ssRNA genome, 2.3–3.6 kb in size, that encodes the protein RNA-dependent RNA polymerase (RdRp) [13]. Since both the coat protein (CP) and movement protein (MP) are lacking in the narnaviruses, their genomes exist as RNA/RdRp nucleoprotein complexes within lipid vesicles in the cells. From RdRp-based phylogenetic analysis, the narnaviruses are closely related to plant viruses in the genus of *Ourmiavirus*, whose genome is composed of three ssRNA segments encoding proteins of RdRp, CP, and MP [14]. It has been proposed that the viruses of the *Ourmiavirus* genus are recombinants of narnavirus-like viruses and plant viruses, such as tombusviruses [14]. The CP and MP of ourmiaviruses are similar to other plant viruses, whereas the RdRps are closely related to narnaviruses.

*Magnaporthe oryzae* is the causal agent of rice blast, the most destructive disease of rice that could result in great yield loss in rice fields annually. Polyhedral virus particles suggestive of virus infection were previously reported in this fungus, the first plant-pathogenic fungus from which viruses were isolated [15,16]. Recently, a few mycoviruses were isolated and identified in this pathogenic fungus. Among these, three distinct viruses, named *Magnaporthe oryzae* virus 1, 2, and 3 (MoV1, MoV2, and MoV3) were assigned to the family Totiviridae and two viruses, *Magnaporthe oryzae* chrysovirus 1A and B (MoCV1-A and MoCV1-B) were assigned to the family Chrysoviridae. The MoCV1-A and MoCV1-B have the ability to impair vegetative and invasive growth, as well as to alter the colony morphology of their host fungus [17,18,19]. In addition, other (+)ssRNA viruses have also been described: *Magnaporthe oryzae* virus A (MoVA) [20] and *Magnaporthe oryzae* ourmia-like virus 1 (MOLV1) [21]. The RdRps encoded by MoVA and MOLV1 are similar to those observed in plant viruses of the Tombusviridae family and *Ourmiavirus* genus, respectively.

In this study, we characterized a novel mycovirus, named *Magnaporthe oryzae* ourmia-like virus 4 (MOLV4), from a *M. oryzae* strain HNDW-6. Viral genome organization and phylogenetic analysis indicate that this virus is closely related to the ourmia-like mycoviruses and the plant viruses of the *Ourmiavirus* genus. Bioinformatic analyses led to the finding that related viral sequences were present in the database, such as the transcriptome shotgun libraries (TSA) and expressed sequence tag database (ESTdb), suggesting a wide prevalence of these kinds of viruses across kingdoms. In addition, biological function of the MOLV4 have been discussed.

## 2. Materials and Methods

### 2.1. Fungal Isolates and Growth Conditions

*M. oryzae* strain HNDW-6 was originally isolated from rice leaves infected with rice blast disease in Hunan province, China. It was grown on potato dextrose agar (PDA; potato, glucose, agarose) at 27 °C. For dsRNA extraction, mycelial plugs were cultured in potato dextrose (PD) broth (potato, glucose) liquid medium at 27 °C, in an orbital shaker at 110 rpm for 4 to 7 days.

### 2.2. DsRNA Extraction and Purification

DsRNAs were extracted from fungal mycelium using the methods described by Morris and Dodds with minor modifications [22]. DNA and ssRNA contaminants were eliminated by digestion with RNase-free DNase I and S1 nuclease (TaKaRa, Dalian, China). The extracted dsRNAs were fractionated by agarose gel (1%, *w*/*v*) electrophoresis and visualized under an AlphaImager HP gel imaging system (ProteinSimple, Silicon Valley, San Francisco, CA, USA) after being stained in a 0.1 mg/mL ethidium bromide solution.

### 2.3. cDNA Cloning and Sequencing

The dsRNAs were purified and used as templates for cDNA synthesis. cDNA libraries were constructed using random hexadeoxynucleotide primers (TaKaRa, Dalian, China) based on the methods described previously [23]. The internal gap regions of the viral genome were filled by RT-PCR amplification using sequence-specific primers designed based on obtained sequences (F:5′-TCTCGTTGCCTTGATGTCGT-3′/R:5′-GACCACGAGACCGACCAAAG-3′). Using this primer pair, a PCR product in size of 300 bp would be obtained. To obtain the terminal sequence of the dsRNA, a ligase-mediated terminal amplification method was used. All the amplified DNA fragments were purified, cloned, and Sanger sequenced. Every base was determined by sequencing at least three independent overlapping clones.

### 2.4. Database Search

Database searches were conducted against the EST and TSA databases available from NCBI using the tBLASTn, as described previously [24]. For this search, the amino acid sequence of MOLV4 was used as a query, and the sequences of plant and fungal transcriptome that matched viral protein with E-values of <0.01 were extracted. The collected sequences were then assembled and used to screen the non-redundant (NR) database with a reciprocal BLASTX.

### 2.5. Sequence Analysis

Potential open reading frames (ORFs) of the full-length cDNA sequence were deduced and their homologous amino acid sequences were searched in NCBI by ORF Finder and BLASTp programs, respectively. Multiple sequence alignment was carried out using the CLUSTALX 1.8 program [25]. Phylogenetic analysis was carried out with the neighbor-joining (NJ) method in MEGA 7 programs [26]. Bootstrap values supporting the phylogenetic tree were calculated after 1000 re-samplings. Potential secondary structures at the 5′ and 3′ terminal sequences of the viral genome sequence were predicted using the Mfold with DINAMeltWeb Server (http://mfold.rna.albany.edu/?q=DINAMelt/Quickfold) [27].

### 2.6. Elimination of the Mycovirus

In order to determine the effects of the virus infection on the host fungus, we used compounded methods of thermal treatment and cycloheximide to eliminate the virus from strain HNDW-6 as described previously with minor modifications [28]. Hyphal tips of strain HNDW-6 were transferred to PDA medium containing cycloheximide with concentrations of 0.5, 0.75, and 1.0 μg/mL. The treatments of each concentration had three replicates and were incubated at 32 °C for 7 days. Then, hyphal tips were transferred onto another set of PDA medium containing the same concentrations of cycloheximide for 7 days. After three successive treatments, hyphal tips were sub-cultured on PDA without cycloheximide at 27 °C for 7 days. Total RNAs was extracted and RT-PCR was used to detect the presence of virus using the primers (MOLV4-F: 5′-ATGTGAAGGTGGTATGTTGGT-3′/MOLV4-R: 5′-CTACTCGTTCTGAGATGTAGCG-3′).

### 2.7. Biological Assessment

Morphology and growth rates were assessed by culturing mycelial plugs, collected from the 4-day-old actively growing plate, on PDA for 3–7 days at 27 °C in the dark. Mycelial growth rates were tested by measuring the diameters of each colony. The test of each strain had three repetitive PDA plates. The data for the growth rate test were analyzed by a one-way analysis of variance in the SPSS software (IBM SPSS statistics 20). Treatment means of mycelial growth rates between different fungal stains were compared using Student’s *t* test at α = 0.05.

## 3. Results

### 3.1. Discovery of a Mycovirus in M. oryzae Strain HNDW-6

**Figure 1:**
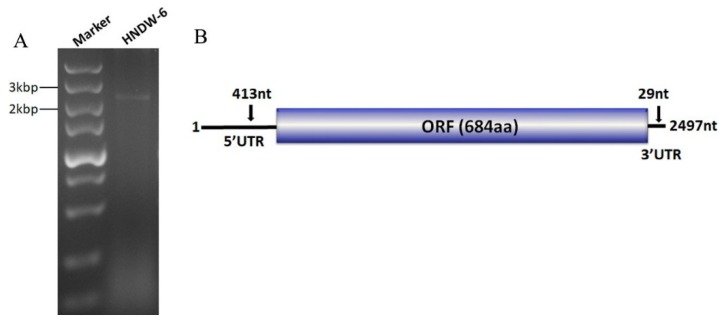
Agarose gel electrophoresis analysis of the dsRNA extracted from the *Magnaporthe oryzae* strain HNDW-6 and the genome organization of MOLV4. (**A**) dsRNA pattern of strain HNDW-6 was observed on 1% agarose gel, with the size estimated with a DNA marker. (**B**) Schematic representation of the genome of MOLV4, which contained a single open reading frame (ORF) putatively encoding the RNA-dependent RNA polymerase (RdRp) and the 5′and 3′ untranslated regions (UTRs).

In an attempt to screen mycoviruses from *M. oryzae* strains, a dsRNA segment of approximately 2–3 kb in size was detected from the strain HNDW-6 when extracted with CF-11 cellulose chromatography method and electrophoresed on 1% agarose gel (Figure 1A). The dsRNA nature of the nucleic acids extracts was confirmed since they were resistant to digestion of DNase I and S1 nuclease.

### 3.2. Cloning and Sequence Analysis of the Mycovirus Associated with Strain HNDW-6

The sequence of the dsRNA was deposited in the Genbank under the accession number MK507958. Sequence analysis indicated that this dsRNA was the genome of a novel (+)ssRNA mycovirus named MOLV4. The genome organization of this virus is illustrated in Figure 1B.

**Figure 2:**
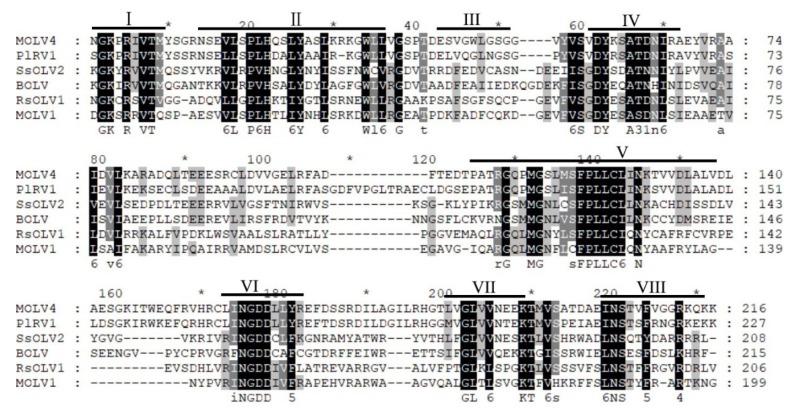
Multiple alignment of the amino acid sequences of RdRp encoded by MOLV4 and other related ourmia-like viruses. The conserved motifs in RdRp of the compared viruses are shown by roman numerals I to VIII. Abbreviations: PIRV1, *Phomopsis longicolla* RNA virus 1; SsOLV2, *Sclerotinia sclerotiorum* ourmia-like virus 2; BOLV, *Botrytis* ourmia-like virus; RsOLV1, *Rhizoctonia solani* ourmia-like virus 1; MOLV1, *Magnaporthe oryzae* ourmia-like virus 1. Identical residues are colour-highlighted with black shadow, and conserved and semi-conserved amino acid residues are colour-highlighted with gray shadow.

The full-length cDNA of MOLV4 was 2497 bp long, with a G + C content of 54.8%. It contained a single ORF that initiated at the nucleotide position 414 and terminated at position 2468. The ORF was predicted to encode a polypeptide of 684 amino acid (aa) residues with a calculated molecular weight of 76.7 kDa. This protein showed 57% (E-value: 0; query cover: 99%) aa identity to the RdRp of *Phomopsis longicolla* RNA virus 1 (PlRV1), followed by other ourmia-like mycoviruses, including *Sclerotinia sclerotiorum* ourmia-like virus 3 (SsOLV3) (E-value: 1e–22; query cover: 64%; identity: 25%), *Rhizoctonia solani* ourmia-like virus 1 (RsOLV1) (E-value: 4e–19; query cover: 39%; identity: 30%), *Botrytis* ourmia-like virus (OuMV) (E-value: 2e–18; query cover: 57%; identity: 27%), and other plant ourmiaviruses (Table 1). Conserved domain database (CDD) search and multiple sequences alignment showed that the MOLV4 encoded protein contained the conserved motifs characteristic of the viral RdRps of (+)ssRNA viruses [29] (Figure 2).

### 3.3. 5′- and 3′-Untranslated Regions (UTRs) of MOLV4

**Figure 3:**
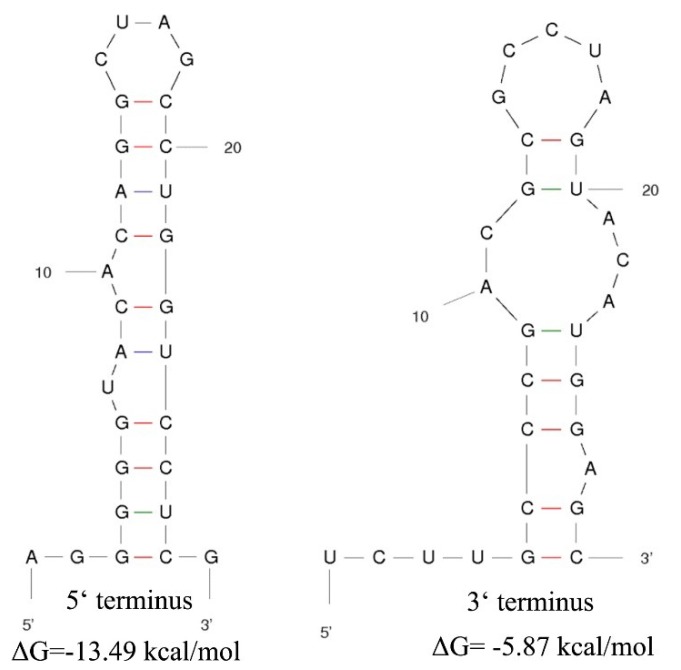
Potential secondary structures of the 5′ and 3′ termini of MOLV4. The putative stem-loop structures are depicted and the ΔG values (kcal/mol) were calculated using RNAfold program. Short lines in different colours indicate hydrogen bonds between different base pairs (red: G-C pairs; purple: A-U pairs; green: G-U pairs).

The genome of MOLV4 had a 5′-UTR of 413 bp and a 3′-UTR of 29 bp. The potential secondary structures of the termini of the positive-strand MOLV4 were predicted using Mfold RNA structure software [27]. The first 24 nt of the 5′-UTR could be folded into a stable stem-loop structure with a ΔG value of –13.49 kcal/mol, while the 3′-UTRs were predicted to form a stem-loop structure with a ΔG value of –5.87 kcal/mol (Figure 3).

### 3.4. Phylogenetic Analysis of MOLV4 and Their Relatives

**Figure 4:**
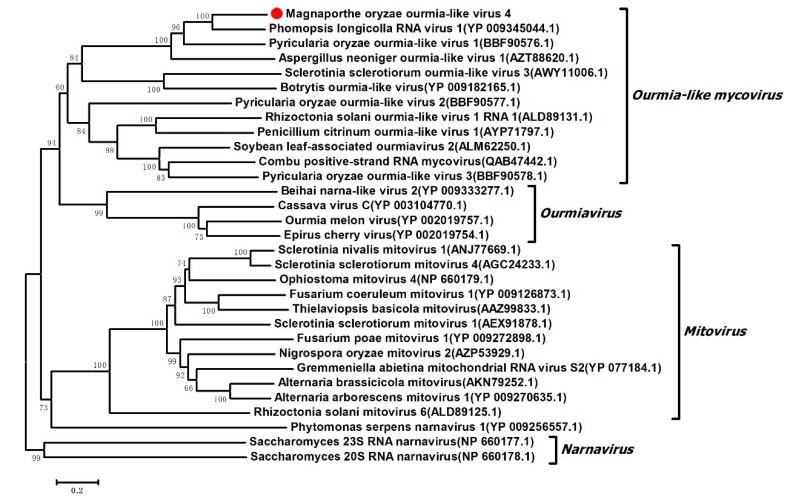
Phylogenetic analysis of MOLV4. The phylogenetic tree was constructed based on the alignment of aa sequences of RdRp using the neighbor-joining method. The bootstrap values were calculated with 1000 replicates. Bootstrap values are shown next to the branches. MOLV4 is indicated red dot in the phylogenetic tree and the substitutions per nucleotide position are represented by scale bars.

To elucidate the evolutionary history of MOLV4, phylogenetic analysis was performed using the full-length aa sequence of the RdRp encoded by MOLV4 and other selected viral sequences, including those of the ourmia-like mycoviruses and the viruses in the Narnaviridae family or the *Ourmiavirus* genus. These aa sequences were multiple aligned and used for phylogenetic tree generation by the NJ method in MEGA 7 [26]. As expected from BLAST search results, the phylogenetic tree revealed that MOLV4 was grouped with the ourmia-like mycoviruses, such as the PlRV1, SsOLV3, BOLV, and RsOLV1. These viruses formed a clade closely related to the plant viruses in the genus *Ourmiavirus*, but distinct from the mycoviruses in the family Narnaviridae (Figure 4). Based on the phylogenetic analyses, we supposed that MOLV4 was a new ourmia-like mycovirus, which could be placed in a putative genus “*Ourmycovirus*” as proposed previously [30].

### 3.5. Searching for MOLV4-Like Sequences in Database

Homology searches of the public database such as the genomes and transcriptomes of cellular organisms have been reported to be an available approach for the detection of novel viral sequences and other unraveled non-retroviral RNA and viruses integrated into eukaryotic genomes [24,31,32,33,34]. In this study, we searched for the presence of MOLV4-like sequences in the TSA database at GenBank using the deduced MOLV4 encoded protein as query. The efforts yielded several significant matches in the fungi (*Monilinia fructicola*, *Podosphaera xanthii*, *Sclerotinia homoeocarpa*, and *Leucocoprinus* sp.), plants (*Chionochloa macra*, *Triticum aestivum*, *Saccharum*, *Araucaria cunninghamii*, *Abies pinsapo*, *Gleditsia sinensis*, *Phlox roemeriana*, *Datisca glomerate*, *Dactylorhiza fuchsia*, *Agrostis stolonifera*, *Salix integra*, *Helianthus niveus*, *Suaeda fruticose*, *Actinidia deliciosa*, *Nepenthes khasiana*, *Cymbidium faberi*, *Humulus lupulus*), vertebrates (*Sardina pilchardus*), and arthropods (*Ixodes scapularis*, *Unaspis euonymi*, *Loxostege sticticalis*). Matches from the TSA libraries of fungi, plants, vertebrata, and arthropods are listed in Table S1.

In addition, we searched the EST database for MOLV4-like sequences. We found 100 MOLV4 related accessions derived from the EST libraries in several fungal strains including *Botryotinia fuckeliana*, *B. tulipae*, *Magnaporthe grisea*, and *Blumeria graminis*. The accessions were then assembled into seven MOLV4-like sequences (contigs or singletons) i.e., MoESTc1_OL and MoESTc2_OL from *M. grisea*, BtESTc1_OL and BtESTc2_OL from *B. tulipae*, BfESTc1_OL from *B. fuckeliana*, and BgESTc1_OL and BgESTc2_OL from *B. graminis*. Reverse BLAST analysis of the viral-like EST sequences on the complete NR database revealed 30% to 86% aa identities to the RdRps of ourmia-like mycoviruses and ourmiaviruses.

Interestingly, using the MOLV4 sequences as a query, we also found two MOLV4 like sequences in the GSS library by a tBLASTn search. The two GSS sequences from the *Symbiodinium minutum* genomic clone SYB2F-146E05 (GA560351) and the uncultured marine RNA virus genomic (DX421080.1) showed moderate levels of aa sequence identities (26% and 29%, respectively).

Several previous studies have demonstrated the presence of non-retroviral RNA viruses and DNA viruses in the eukaryotic genome. During BLAST searching for the MOLV4 like sequences in the whole-genome shotgun (WGS) library, three significant matches of chromosomes of the *Calonectria naviculata* (NAGG01000037.1), *C. leucothoes* (NAJI01000289.1) and *C. pseudonaviculata* (JYJY01001043.1) were found, which were termed as *Calonectria naviculata* MOLV4-like sequence 1 (CnMLS1), *Calonectria leucothoes* MOLV4-like sequence 1 (ClMLS1), and *Calonectria pseudonaviculata* MOLV4-like sequence 1 (CpMLS1). BLASTX searches in the NR database, using the three hit sequences as queries, revealed 24% to 55% aa sequence identities to the ourmia-like viruses.

### 3.6. Influence of MOLV4 on M. oryzae

**Figure 5:**
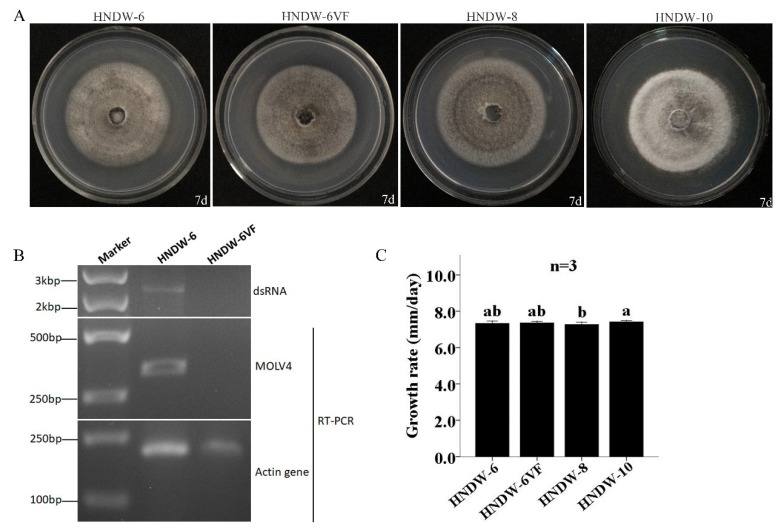
Virus elimination and biological comparison between the virus-containing strain HNDW-6 and the isogenic virus-cured strain HNDW6-VF. (**A**) Colony morphology of HNDW-6, HNDW6-VF, HNDW-8, and HNDW-10 on PDA medium at 27 °C for 7 days. (**B**) Detection of MOLV4 using dsRNA extraction and RT-PCR methods. Lane Marker, DNA marker. (**C**) Growth rates of HNDW-6, HNDW6-VF, HNDW-8, and HNDW-10 on PDA medium at 27 °C. The bars indicate standard deviation from three sample means and the letter indicates that there was no significant difference at α = 0.05 level of confidence.

The thermal and cycloheximide treatments were used together to eliminate MOLV4 from the strain HNDW-6. An isogenic MOLV4-free derivative isolate, named HNDW-6VF, was obtained and confirmed by RT-PCR. The virus-carrying HNDW-6, virus-free HNDW-6VF, and two other reference virus-free *M. oryzae* strains (HNDW-8 and HNDW-10) were compared in biological traits including colony morphology and growth rate. Aside from the HNDW-10, which has a relative lighter color, the HNDW-6, HNDW-6VF, and the HNDW-8 showed similar morphological characteristics. In addition, the four *M. oryzae* strains had no significant difference in growth rate. Strains HNDW-6, HNDW-6VF, HNDW-8, and HNDW-10 grew at the average rates of 7.33, 7.36, 7.27, and 7.41 mm/d, respectively (Figure 5). These results showed that the MOLV4 caused an asymptomatic infection in its host, at least in terms of morphology and growth rate.

## 4. Discussion

In this study, we identified a novel mycovirus, MOLV4, isolated from the plant pathogenic fungus *M. oryzae*. MOLV4 encoded only a single protein of RdRp sharing aa sequence identities to the RdRps of ourmia-like mycoviruses, which were more similar to the plant ourmiaviruses rather than to mycoviruses in the Narnaviridae family. Based on the genome organization, homology search, and phylogenetic analysis, MOLV4 was proposed to be a novel mycovirus closely related to ourmiaviruses. Virus elimination and biological comparison indicated that the MOLV4 infection had no or mild effects on the phenotypes of the host. Illana et al. [21] reported an ourmia-like mycovirus, MOLV1, in *M. oryzae*. MOLV4 was the second reported ourmia-like mycovirus infecting *M. oryzae.* However, the RdRp of MOLV4 shared the maximal aa identity of 57% with that of PlRV1, but had a very low degree of identity to that of MOLV1. The result indicated that the ourmia-like mycoviruses might be widely distributed and ancestrally present among the fungal hosts.

Since the ourmia-like mycoviruses were similar to the ourmiaviruses and narnaviruses according to RdRp-based phylogenetic analysis, elucidating the origin and evolution of the ourmia-like mycoviruses will have important implications in the understanding of the complex relationship between fungal narnaviruses and plant ourmiaviruses. Previous studies proposed that the ourmiaviruses might have evolved from a narnavirus (the fungal virus that encodes only the RdRp protein) via acquisition of the genome segments, encoding MP and CP, from a plant virus, tombusvirus [14]. However, Ghabrial et al. [35] hypothesized that mycoviruses might also have evolved from plant viruses through the loss of genes encoding proteins dispensable for their survival inside the fungal host. Actually, some mycoviruses have been reported to share important similarity with plant viruses. For example, a *Sclerotinia sclerotiorum* debilitation-associated RNA virus, identified from the *Sclerotinia sclerotiorum*, was reported to be closely related to the allexiviruses in the family Flexiviridae [36]. The *Diaporthe ambigua* RNA virus [37] and MoVA [20], which infected the *Diaporthe ambigua* and *Magnaporthe oryzae*, respectively, were also reported to share distinct relatedness to plant viruses of the family Tombusviridae. The difference between these mycoviruses and their related plant viruses is that the former lacks coding sequences for CP and MP. Since the mycoviruses could be transmitted via hyphal anastomosis with cytoplasmic flow, some structure proteins, at least the MP, seemed to be unnecessary for survival of the mycoviruses. Even in plant viruses, a CP mutant of the turnip crinkle virus (TCV) still has the ability to replicate in cells of plant leaves [38]. In addition, natural deletion of viral genes in some cases might occur in virus hosts. Examples of CP deletion were previously found in some plant viruses, including the benyviruses and other viruses that are normally vectored by the fungi *Olpidium*, *Polymyxa*, or *Spongospora*. This appeared when these viruses were transmitted repeatedly between plants without the involvement of fungi or protists [39,40,41]. Therefore, it is also possible that the ourmia-like mycoviruses might have originated from ourmiavirus infecting the plant host and later adapted to the fungal host by losing the dispensable CP and MP, as has been suggested previously [42]. Of course, additional model studies with long-term monitoring of the virus adaptation and evolution within fungal hosts is still required to verify the evolution history of the ourmia-like mycoviruses.

Actually, studying the evolution of some viruses is complex, as the presence of virus transmission between different hosts was considered to be important power for virus evolution. Nerva et al. [43] reported that some mycoviruses of endophytic fungi that belong to the families of Partitiviridae and Totiviridae could replicate in plant protoplast cells without evidence of host adaptation. Reciprocally, some experimental evidences indicated that some plant viruses could replicate in fungi cells. For example, brome mosaic virus (BMV) has been demonstrated experimentally to replicate in yeast, *Saccharomyces cerevisiae* [44] and later, the replication of tobacco mosaic virus (TMV) in phytopathogenic fungus *Colletotrichum acutatum* was also confirmed [45]. Recently, a naturally occurring plant virus, cucumber mosaic virus (CMV), was reported to infect the phytopathogenic fungus *Rhizoctonia solani*, providing valid evidence for the transfer of a plant virus between plant and fungus [46]. It is known that some plant viruses, such as these in the Tombusviridae family, are transmitted by soil-borne fungi in the genus *Olpidium* or merely through soil without fungal vectors [47,48], while these in the families of Bunyaviridae, Rhabdoviridae, Reoviridae, and genus *Tenuivirus* are transmitted by insect vectors [49,50]. Recently, a fungal virus was even confirmed to infect an insect and use it as a vector [51]. Collectively, the insect and fungus vectors, as well as the common ecological niches used for different viruses and hosts, could provide opportunities for horizontal virus transfer between different taxa. Thus, with more examples of cross-kingdom viral infection found, the more information associated with virus evolution and genetic diversity could be provided.

It is possible that some plant or fungi materials used for cDNA library construction might carry viruses that often render virus detection difficult, thus, it is possible to screen viral-like sequences from the TSA and EST databases. In this study, using the MOLV4 sequence as a query, we found many MOLV4-like sequences in the TSA database of many eukaryotes, including plants, fungi, vertebrata, and arthropods. In addition, several EST sequences from the fungus *B. graminis*, *B. fuckeliana*, *B. tulipae*, and *M. grisea* were found to be similar with the MOLV4 sequences. This indicated that, with the exception of recorded fungus, such as *M. grisea* and *Botrytis* sp., the ourmia-like mycoviruses could infect more host organisms, extending the diversity and possible host range of the ourmia-like mycoviruses. In conclusion, the finding of these new viral sequences will help to provide new insights into the origin and evolution of the ourmia-like viruses. Of course, it is yet to be determined if some of the found viral sequences from plants or the other TSA database are truly derived from these annotated host organisms. Since many plants could carry endophytic fungi, which might be infected by mycoviruses [52], we could not exclude the possibility that some virus-like sequences found in cDNA libraries of plants might actually be mycoviruses derived from the endophytic fungi. On the other hand, the discovery of MOLV4 like sequences from different organisms indicated the potential hosts of these viruses and extended our understanding of the possible host range of the ourmia-like mycoviruses and ourmiaviruses. For example, the ourmia-like viruses have previously been detected only from fungi or plants; the discovery of MOLV4-like sequences in the cDNA libraries of arthropods and vertebrata suggest the possibility that these viruses might infect animals.

## Figures and Tables

**Table 1:** Information of BLASTp search results of the RNA-dependent RNA polymerase (RdRp) of *Magnaporthe oryzae* ourmia-like virus 4 (MOLV4).

Taxon	Virus Name	Abbreviation	Accession	Query Cover (%)	Identity (%)	E-Value
Ourmia-like mycoviruse	Phomopsis longicolla RNA virus 1	PlRV1	YP_009345044.1	99	407/716 (57%)	0
	Pyricularia oryzae ourmia-like virus 1	POLV1	BBF90576.1	94	299/654 (46%)	5e–162
	Aspergillus neoniger ourmia-like virus 1	AnOLV1	AZT88620.1	78	245/578 (42%)	2e–124
	Sclerotinia sclerotiorum ourmia-like virus 3	SsOLV3	AWY11006.1	64	123/496 (25%)	1e–22
	Rhizoctonia solani ourmia-like virus 1	RsOLV1	ALD89131.1	39	91/304 (30%)	4e–19
	Combu positive-strand RNA mycovirus	CRMV	QAB47442.1	30	78/218 (36%)	7e–19
	Sclerotinia sclerotiorum ourmia-like virus 2	SsOLV2	ALD89139.1	38	93/268 (35%)	1e–18
	Botrytis ourmia-like virus	BOLV	YP_009182165.1	57	119/433 (27%)	2e–18
	Sclerotinia sclerotiorum ourmia-like virus 1	SsOLV1	ALD89138.1	46	103/341 (30%)	7e–17
	Soybean leaf-associated ourmiavirus 1	SlaOMV1	ALM62238.1	53	117/406 (29%)	3e–16
	Penicillium citrinum ourmia-like virus 1	PcOLV1	AYP71797.1	33	72/230 (31%)	6e–15
	Soybean leaf-associated ourmiavirus 2	SlaOMV2	ALM62250.1	53	114/419 (27%)	7e–15
*Ourmiavirus*	Cassava virus C	CaV-C	YP_003104770.1	23	56/180 (31%)	3e–05
	Ourmia melon virus	OmV	YP_002019757.1	22	53/178 (30%)	4e–05
	Epirus cherry virus	EcV	YP_002019754.1	23	54/176 (31%)	7e–05

## Supplementary Materials

The following are available online at https://www.mdpi.com/1999-4915/11/3/223/s1, Table S1: MOLV4-like sequences obtained from the NCBI TSA, EST, GSS and WGS database.

## References

[1] Ghabrial S.A., Suzuki N. (2009). Viruses of plant pathogenic fungi. Annu. Rev. Phytopathol..

[2] Jian J., Lakshman D.K., Tavantzis S.M. (1997). Association of distinct double-stranded RNAs with enhanced or diminished virulence in *Rhizoctonia solani* infected potato. Mol. Plant Microbe Interact..

[3] Ahn I.P., Lee Y.H. (2001). A viral double-stranded RNA up regulates the fungal virulence of *Nectria radicicola*. Mol. Plant Microbe Interact..

[4] Park C.M., Banerjee N., Koltin Y., Bruenn J.A. (1996). The Ustilago maydis virally encoded KP1 killer toxin. Mol. Microbiol..

[5] Schmitt M.J., Breinig F. (2006). Yeast viral killer toxins: Lethality and self-protection. Nat. Rev. Microbiol..

[6] Márquez L.M., Redman R.S., Rodriguez R.J., Roossinck M.J. (2007). A Virus in a Fungus in a Plant: Three-Way Symbiosis Required for Thermal Tolerance. Science.

[7] Ghabrial S.A., Castón J.R., Jiang D., Nibert M.L., Suzuki N. (2015). 50-plus years of fungal viruses. Virology.

[8] Xie J., Jiang D. (2014). New insights into mycoviruses and exploration for the biological control of crop fungal diseases. Annu. Rev. Phytopathol..

[9] Nuss D.L. (1992). Biological control of chestnut blight: An example of virus-mediated attenuation of fungal pathogenesis. Microbiol Rev..

[10] Yu X., Li B., Fu Y., Jiang D., Ghabrial S.A., Li G., Peng Y., Xie J., Cheng J., Huang J. (2010). A geminivirus-related dna mycovirus that confers hypovirulence to a plant pathogenic fungus. Proc. Natl. Acad. Sci. USA.

[11] Liu L., Xie J., Cheng J., Fu Y., Li G., Yi X., Jiang D. (2014). Fungal negative-stranded RNA virus that is related to bornaviruses and nyaviruses. Proc. Natl. Acad. Sci. USA.

[12] Cai G., Myers K., Fry W.E., Hillman B.I. (2012). A member of the virus family Narnaviridae from the plant pathogenic oomycete *Phytophthora infestans*. Arch. Virol..

[13] Hillman B.I., Cai G. (2013). The family *Narnaviridae*: Simplest of RNA viruses. Adv. Virus Res..

[14] Rastgou M., Habibi M.K., Izadpanah K., Masenga V., Milne R.G., Wolf Y.I., Turina M. (2009). Molecular characterization of the plant virus genus *Ourmiavirus* and evidence of inter-kingdom reassortment of viral genome segments as its possible route of origin. J. Gen. Virol..

[15] Hunst P.L., Latterell F.M., Rossi A.E. (1986). Variation in double-stranded RNA from isolates of *Pyricularia oryzae*. Phytopathology.

[16] Yamashita S., Doi Y., Yora K. (1971). A polyhedral virus found in rice blast fungus, *Pyricularia oryzae* Cavara. Jpn. J. Phytopathol..

[17] Urayama S., Kato S., Suzuki Y., Aoki N., Le M.T., Arie T., Moriyama H. (2010). Mycoviruses related to chrysovirus affect vegetative growth in the rice blast fungus *Magnaporthe oryzae*. J. Gen. Virol..

[18] Urayama S., Ohta T., Onozuka N., Sakoda H., Fukuhara T., Arie T., Moriyama H. (2012). Characterization of Magnaporthe oryzae chrysovirus 1 structural proteins and their expression in *Saccharomyces cerevisiae*. J. Virol..

[19] Urayama S.I., Sakoda H., Takai R., Katoh Y., Le T.M., Fukuhara T., Moriyama H. (2014). A dsRNA mycovirus, *Magnaporthe oryzae* chrysovirus 1-B, suppresses vegetative growth and development of the rice blast fungus. Virology.

[20] Ai Y.P., Zhong J., Chen C.Y., Zhu H.J., Gao B.D. (2016). A novel single-stranded RNA virus isolated from the rice-pathogenic fungus *Magnaporthe oryzae* with similarity to members of the family *Tombusviridae*. Arch. Virol..

[21] Illana A., Marconi M., Rodríguez-Romero J., Xu P., Dalmay T., Wilkinson M.D., Sesma A. (2017). Molecular characterization of a novel ssRNA ourmia-like virus from the rice blast fungus *Magnaporthe oryzae*. Arch. Virol..

[22] Morris T.J., Dodds J.A. (1979). Isolation and analysis of double-stranded RNA from virus-infected plant and fungal tissue. Phytopathology.

[23] Zhong J., Pang X.D., Zhu H.J., Gao B.D., Huang W.K., Zhou Q. (2016). Molecular characterization of a trisegmented mycovirus from the plant pathogenic fungus *Colletotrichum gloeosporioides*. Viruses.

[24] Kondo H., Chiba S., Toyoda K., Suzuki N. (2013). Evidence for negative-strand RNA virus infection in fungi. Virology.

[25] Thompson J.D., Gibson T.J., Plewniak F., Jeanmougin F., Higgins D.G. (1997). The CLUSTALX windows interface: Flexible strategies for multiple sequence alignment aided by quality analysis tools. Nucleic Acids Res..

[26] Kumar S., Stecher G., Tamura K. (2016). MEGA7: Molecular evolutionary genetics analysis version 7.0 for bigger datasets. Mol. Biol. Evol..

[27] Zuker M. (2003). Mfold web server for nucleic acid folding and hybridization prediction. Nucleic Acids Res..

[28] Ejmal M.A., Holland D.J., MacDiarmid R.M., Pearson M.N. (2018). A novel chrysovirus from a clinical isolate of Aspergillus thermomutatus affects sporulation. PLoS ONE.

[29] Koonin E.V. (1991). The phylogeny of RNA-dependent RNA polymerases of positive-strand RNA viruses. J. Gen. Virol..

[30] Hrabáková L., Koloniuk I., Petrzik K. (2017). Phomopsis longicolla RNA virus 1–Novel virus at the edge of myco-and plant viruses. Virology.

[31] Liu H., Fu Y., Jiang D., Li G., Xie J., Cheng J., Yi X. (2010). Widespread horizontal gene transfer from double-stranded RNA viruses to eukaryotic nuclear genomes. J. Virol..

[32] Liu H., Fu Y., Xie J., Cheng J., Ghabrial S.A., Li G., Jiang D. (2012). Evolutionary genomics of mycovirus-related dsRNA viruses reveals cross-family horizontal gene transfer and evolution of diverse viral lineages. BMC Evol. Biol..

[33] Chiba S., Kondo H., Tani A., Saisho D., Sakamoto W., Kanematsu S., Suzuki N. (2011). Widespread endogenization of genome sequences of non-retroviral RNA viruses into plant genomes. PLoS Pathog..

[34] Zhang R., Liu S., Chiba S., Kondo H., Kanematsu S., Suzuki N. (2014). A novel single-stranded RNA virus isolated from a phytopathogenic filamentous fungus, *Rosellinia necatrix*, with similarity to hypo-like viruses. Front Microbiol..

[35] Ghabrial S.A. (1998). Origin, adaptation and evolutionary pathways of fungal viruses. Virus Genes.

[36] Xie J., Wei D., Jiang D., Fu Y., Li G., Ghabrial S., Peng Y. (2006). Characterization of debilitation-associated mycovirus infecting the plant-pathogenic fungus *Sclerotinia sclerotiorum*. J. Gen. Virol..

[37] Preisig O., Moleleki N., Smit W.A., Wingfield B.D., Wingfield M.J. (2000). A novel RNA mycovirus in a hypovirulent isolate of the plant pathogen *Diaporthe ambigua*. J. Gen. Virol..

[38] Hacker D.L., Petty I.T.D., Wei N., Morris T.J. (1992). Turnip crinkle virus genes required for RNA replication and virus movement. Virology.

[39] Rossi M., Vallino M., Abbà S., Ciuffo M., Balestrini R., Genre A., Turina M. (2015). The importance of the KR-rich region of the coat protein of Ourmia melon virus for host specificity, tissue tropism, and interference with antiviral defense. Mol. Plant Microbe Interact..

[40] Reavy B., Arif M., Cowan G.H., Torrance L. (1998). Association of sequences in the coat protein/readthrough domain of potato mop-top virus with transmission by *Spongospora subterranea*. J. Gen. Virol..

[41] Kakani K., Sgro J.Y., Rochon D.A. (2001). Identification of specific cucumber necrosis virus coat protein amino acids affecting fungus transmission and zoospore attachment. J. Virol..

[42] Marzano S.Y.L., Nelson B.D., Ajayi-Oyetunde O., Bradley C.A., Hughes T.J., Hartman G.L., Domier L.L. (2016). Identification of diverse mycoviruses through metatranscriptomics characterization of the viromes of five major fungal plant pathogens. J. Virol..

[43] Nerva L., Varese G.C., Falk B.W., Turina M. (2017). Mycoviruses of an endophytic fungus can replicate in plant cells: Evolutionary implications. Sci. Rep..

[44] Janda M., Ahlquist P. (1993). RNA-dependent replication, transcription, and persistence of brome mosaic virus RNA replicons in *S. cerevisiae*. Cell.

[45] Mascia T., Nigro F., Abdallah A., Ferrara M., De Stradis A., Faedda R., Gallitelli D. (2014). Gene silencing and gene expression in phytopathogenic fungi using a plant virus vector. Proc. Natl. Acad. Sci. USA.

[46] Andika I.B., Wei S., Cao C., Salaipeth L., Kondo H., Sun L. (2017). Phytopathogenic fungus hosts a plant virus: A naturally occurring cross-kingdom viral infection. Proc. Natl. Acad. Sci. USA.

[47] Knowles N.J., Hovi T., Hyypia T., King A.M.Q., Lindberg A.M., Pallansch M.A., Yamashita T. (2012). Virus Taxonomy: Eighth Report of the International Committee on Taxonomy of Viruses.

[48] Knowles N.J., Hovi T., Hyypiä T., King A.M., Lindberg A.M., Pallansch M.A., Palmenberg A.C., Simmonds P., Skern T., Stanway G. (2012). Virus Taxonomy: Classification and Nomenclature of Viruses.

[49] Hogenhout S.A., Ammar E.D., Whitfield A.E., Redinbaugh M.G. (2008). Insect vector interactions with persistently transmitted viruses. Annu. Rev. Phytopathol..

[50] Whitfield A.E., Falk B.W., Rotenberg D. (2015). Insect vector-mediated transmission of plant viruses. Virology.

[51] Liu S., Xie J., Cheng J., Li B., Chen T., Fu Y., Jiang D. (2016). Fungal DNA virus infects a mycophagous insect and utilizes it as a transmission vector. Proc. Natl. Acad. Sci. USA.

[52] Herrero N., Márquez S.S., Zabalgogeazcoa I. (2009). Mycoviruses are common among different species of endophytic fungi of grasses. Arch. Virol..

