# Supplementary material for: Characterization of a Novel Ourmia-Like Mycovirus Infecting Magnaporthe oryzae and Implications for Viral Diversity and Evolution
Source: Viruses. 2019 Mar 5;11(3):223. doi: 10.3390/v11030223 (PMC6465991; doi:10.3390/v11030223)
Supplement: Supplementary file 1 [file viruses-11-00223-s001.pdf]

Table S1: MOLV4-like sequences obtained from the NCBI TSA, EST,GSS and WGS database.

Database	Organism	Contig or accession no.	Length (bp)	Best BLASTX hit	Accession no.	Identity(%)	E value	Query cover
EST	Magnaporthe grisea	DC974923	1417	Pyricularia oryzae ourmia-like virus 2	BBF90577.1	202/235(86%)	6e-126	69
	Magnaporthe grisea	Contig	2136	Pyricularia oryzae ourmia-like virus 3	BBF90578.1	209/422(50%)	2e-129	85
	Botrytis tulipae	Contig-1	1984	Combu positive-strand RNA mycovirus	QAB47442.1	122/360(34%)	2e-43	53
	Botrytis tulipae	Contig-2	1791	Penicillium citrinum ourmia-like virus 1	AYP71797.1	213/480(44%)	1e-101	79
	Botryotinia fuckeliana	Contig	1466	Pyricularia oryzae ourmia-like virus 1	BBF90576.1	214/469(46%)	8e-115	94
	Blumeria graminis	Contig-1	2459	Phomopsis longicolla RNA virus 1	YP_0093450 44.1	82/193(42%)	4e-72	64
	Blumeria graminis	Contig-2	1497	Sclerotinia sclerotiorum ourmia-like virus 2	ALD89139.1	166/352(47%)	5e-86	68
TSA	Agrostis stolonifera	Agrostis_stolonifera_c273 710_g1_i1	203	Pyricularia oryzae ourmia-like virus 1	BBF90576.1	37/66(56%)	5e-15	97
	Araucaria cunninghamii	Araucaria_cunninghamii_ Ref_Hoop_Pine	224	Aspergillus neoniger ourmia-like virus 1	AZT88620.1	36/74(49%)	7e-14	97
	Araucaria cunninghamii	Araucaria_cunninghamii_ Ref_Hoop_Pine_	538	Aspergillus neoniger ourmia-like virus 1	AZT88620.1	105/179(59%)	2e-61	99
	Chionochloa macra	Chionochloa_macra_c331 64_g1_i1	2534	Aspergillus neoniger ourmia-like virus 1	AZT88620.1	276/660(42%)	8e-130	73
	Chionochloa macra	Chionochloa_macra_c140 575_g1_i1	2156	Pyricularia oryzae ourmia-like virus 2	BBF90577.1	168/350(48%)	2e-88	48
	Chionochloa macra	Chionochloa_macra_c327 464_g1_i2	2486	Magnaporthe oryzae ourmia-like virus	SBQ28480.1	213/594(36%)	3e-72	70
	Chionochloa macra	Chionochloa_macra_c429 594_g1_i1	873	Magnaporthe oryzae ourmia-like virus	SBQ28480.1	142/293(48%)	6e-70	100
	Cyathus bulleri	Cyathus_bulleri_Transcript _6240	3685	Agaricus bisporus virus 14	AQM49944.1	213/696(31%)	5e-84	54
	Dactylorhiza fuchsii	Dactylorhiza_fuchsii_fB18 55_comp168297_c0_seq1	1726	Combu positive-strand RNA mycovirus	QAB47442.1	112/284(39%)	2e-36	47
	Datisca glomerata	Datisca_glomerata_c22499 _g1_i1	1324	Pyricularia oryzae ourmia-like virus 2	BBF90577.1	200/353(57%)	8e-118	77
	Helianthus niveus	Helianthus niveus	1995	Sclerotinia sclerotiorum ourmia-like virus 2	ALD89139.1	174/376(46%)	2e-79	53

	Sampletranscript17436						
Humulus lupulus	Humulus lupulus var. lupulus mRNA, contig: comp80462_c0_seq1	846	Aspergillus neoniger ourmia-like virus 1	AZT88620.1	160/289(55%)	3e-89	99
Humulus lupulus	Humulus lupulus var. lupulus mRNA, contig: comp268420_c0_seq1	893	Aspergillus neoniger ourmia-like virus 1	AZT88620.1	166/298(56%)	2e-95	97
Humulus lupulus	Humulus lupulus var. lupulus mRNA, contig: comp278983_c0_seq1	397	Pyricularia oryzae ourmia-like virus 1	BBF90576.1	76/132(58%)	3e-43	99
Humulus lupulus	Humulus lupulus var. lupulus mRNA, contig: comp293919_c0_seq1	2572	Aspergillus neoniger ourmia-like virus 1	AZT88620.1	244/516(47%)	2e-130	59
Humulus lupulus	Humulus lupulus var. lupulus mRNA, contig: comp296725_c0_seq6	2652	Aspergillus neoniger ourmia-like virus 1	AZT88620.1	240/505(48%)	1e-138	55
Ixodes scapularis	Ixodes scapularis Isca_Trinity_c595567_g1_i1, c67321_g2_i1	637	Aspergillus neoniger ourmia-like virus 1	AZT88620.1	111/210(53%)	2e-68	97
Ixodes scapularis Isca	Ixodes scapularis Isca_Trinity_c444166_g1_i1	263	Aspergillus neoniger ourmia-like virus 1	AZT88620.1	40/90(44%)	3e-13	99
Leucocoprinus	Leucocoprinus comp14711_c0_seq1	2094	Magnaporthe oryzae ourmia-like virus	SBQ28480.1	124/363(34%)	1e-37	51
Leucocoprinus gongylophorus	Leucocoprinus gongylophorus, isolate Ae322	2131	Magnaporthe oryzae ourmia-like virus	SBQ28480.1	122/363(34%)	1e-38	50
Leucocoprinus sp.	Leucocoprinus sp. HH-2015b comp12727_c0_seq1	1704	Magnaporthe oryzae ourmia-like virus	SBQ28480.1	124/383(32%)	9e-37	41
Leucocoprinus sp.	Leucocoprinus sp. HH-2015b comp13907_c0_seq1	2454	Magnaporthe oryzae ourmia-like virus	SBQ28480.1	124/383(32%)	2e-29	46

Leucocoprinus sp.	Leucocoprinus sp. HH-2015b comp15750_c0_seq1	2626	Magnaporthe oryzae ourmia-like virus	SBQ28480.1	93/226(41%)	7e-37	25
Monilinia fructicola	Monilinia fructicola MFRC_TR_DN6274_c0_g2_i1	2455	Combu positive-strand RNA mycovirus	QAB47442.1	249/516(48%)	5e-129	62
Monilinia fructicola	Monilinia fructicola MFRC_TR_DN9539_c0_g1_i1	2827	Penicillium citrinum ourmia-like virus 1	AYP71797.1	189/488(39%)	1e-63	50
Monilinia fructigena	Monilinia fructigena MFRG_TR_DN8768_c0_g1_i3	3384	Aspergillus neoniger ourmia-like virus 1	AZT88620.1	228/517(44%)	2e-104	44
Nepenthes khasiana	Nepenthes khasiana comp133154_c0_seq1_m_24663	1329	Sclerotinia sclerotiorum ourmia-like virus 2 RNA 1	ALD89139.1	178/450(40%)	2e-88	96
Ophiocordyceps sinensis	Ophiocordyceps sinensis contig09535	1176	Combu positive-strand RNA mycovirus	QAB47442.1	109/272(40%)	2e-40	80
Podosphaera xanthii	Podosphaera xanthii Contig3207	2393	Combu positive-strand RNA mycovirus	QAB47442.1	139/325(43%)	6e-63	39
Podosphaera xanthii	Podosphaera xanthii Contig8947	2058	Magnaporthe oryzae ourmia-like virus	SBQ28480.1	194/493(39%)	9e-88	70
Saccharum	Saccharum_hybrid_cultivar_SUGdenovo-0010839	2802	Penicillium citrinum ourmia-like virus 1	AYP71797.1	246/494(50%)	4e-140	52
Saccharum	Saccharum hybrid cultivar SUGdenovo-0010839	2517	Penicillium citrinum ourmia-like virus 1	AYP71797.1	189/487(39%)	1e-85	56
Saccharum	Saccharum hybrid cultivar SUGdenovo-0063813	2336	Pyricularia oryzae ourmia-like virus 2	BBF90577.1	170/347(49%)	8e-85	44
Saccharum	Saccharum hybrid cultivar SUGdenovo-0201048	883	Soybean leaf-associated ourmiavirus 2	ALM62250.1	125/238(53%)	2e-65	78
Saccharum	Saccharum hybrid cultivar SUGdenovo-0298683	2102	Pyricularia oryzae ourmia-like virus 2	BBF90577.1	189/321(59%)	7e-108	45
Saccharum	Contig	1517	[Aspergillus neoniger ourmia-like virus 1	AZT88620.1	261/398(66%)	4e-170	78
Saccharum	Contig	1423	Phomopsis longicolla RNA virus 1	YP_0093450	267/472(57%)	8e-170	99

				44.1			
Saccharum	Saccharum hybrid cultivar SUGdenovo-0306151	603	Phomopsis longicolla RNA virus 1	YP_0093450 44.1	109/218(50%)	1e-47	98
Saccharum	Saccharum hybrid cultivar SUGdenovo-0307163	426	Combu positive-strand RNA mycovirus	QAB47442.1	83/138(60%)	2e-42	97
Saccharum	Saccharum hybrid cultivar SUGdenovo-0307166	943	Pyricularia oryzae ourmia-like virus 2	BBF90577.1	100/182(55%	1e-50	55
Saccharum	Saccharum hybrid cultivar SUGdenovo-0309206	759	Phomopsis longicolla RNA virus 1	YP_0093450 44.1	65/127(51%)	3e-25	51
Saccharum	Saccharum_hybrid_cultivar_SUGdenovo-0321797，0525616	2328	Combu positive-strand RNA mycovirus	QAB47442.1	277/628(44%)	2e-139	79
Saccharum	Saccharum hybrid cultivar SUGdenovo-0322501	2070	Combu positive-strand RNA mycovirus	QAB47442.1	255/553(46%)	1e-121	79
Saccharum	Saccharum hybrid cultivar SUGdenovo-0323827	2602	Aspergillus neoniger ourmia-like virus 1	AZT88620.1	378/701(54%)	0	77
Saccharum	Saccharum hybrid cultivar SUGdenovo-0461223	2548	Combu positive-strand RNA mycovirus	QAB47442.1	262/563(47%)	1e-125	47
Saccharum	Saccharum hybrid cultivar SUGdenovo-0556108	2468	Penicillium citrinum ourmia-like virus 1	AYP71797.1	196/535(37%)	6e-76	61
Saccharum	Saccharum hybrid cultivar SUGdenovo-0580278	482	Combu positive-strand RNA mycovirus	QAB47442.1	83/146(57%)	6e-39	90
Salix integra	Salix integra comp92875_c0	2658	Penicillium citrinum ourmia-like virus 1	AYP71797.1	207/523(40%)	7e-91	57
Sclerotinia homoeocarpa	Sclerotinia homoeocarpa 7225	820	Pyricularia oryzae ourmia-like virus 1	BBF90576.1	141/262(54%)	3e-79	95
Sclerotinia homoeocarpa	Sclerotinia homoeocarpa 13583	2841	Aspergillus neoniger ourmia-like virus 1	AZT88620.1	248/555(45%)	4e-129	57
Sclerotinia homoeocarpa	Sclerotinia homoeocarpa 14589	1454	Aspergillus neoniger ourmia-like virus 1	AZT88620.1	187/416(45%)	3e-97	83
Sclerotinia homoeocarpa	Sclerotinia homoeocarpa 23242	456	Pyricularia oryzae ourmia-like virus 1	BBF90576.1	90/151(60%)	3e-48	99
Suaeda fruticose (Actinidia	Suaeda fruticose (Actinidia	1374	Combu positive-strand RNA mycovirus	QAB47442.1	208/468(44%)	2e-104	97

	deliciosa)	deliciosa)						
	Suaeda fruticosa	Suaeda fruticosa Locus_57460_Transcript_1/65255	779	Aspergillus neoniger ourmia-like virus 1	AZT88620.1	134/250(54%)	6e-83	93
	Triticum aestivum	Triticum aestivum comp55333_c0	663	Phomopsis longicolla RNA virus 1	YP_009345044.1	109/214(51%)	1e-57	95
	Triticum aestivum	Triticum aestivum comp56567_c0	1064	Sclerotinia sclerotiorum ourmia-like virus 2	ALD89139.1	163/340(48%)	3e-92	95
	Triticum aestivum	Triticum aestivum comp72378_c0	5552	Combu positive-strand RNA mycovirus	QAB47442.1	127/361(35%)	2e-41	38
	Triticum aestivum	Triticum aestivum comp75869_c0	2474	Combu positive-strand RNA mycovirus	QAB47442.1	127/359(35%)	2e-40	41
	Triticum aestivum	Triticum aestivum comp76051_c0	2480	Aspergillus neoniger ourmia-like virus 1	AZT88620.1	245/618(40%)	5e-111	69
	Loxostege sticticalis	Loxostege sticticalis c11542.graph_c0	327	Sclerotinia sclerotiorum ourmia-like virus 1	ALD89138.1	38/96(40%)	9e-09	84
	Loxostege sticticalis	Loxostege sticticalis c72710.graph_c0	242	Sclerotinia sclerotiorum ourmia-like virus 1	ALD89138.1	32/75(43%)	5e-07	88
WGS	Calonectria naviculata	Calonectria naviculata strain CBS 101121 contig_50	657	Combu positive-strand RNA mycovirus	QAB47442.1	94/225(42%)	2e-38	100
	Calonectria leucothoes	Calonectria leucothoes strain CBS 109166 Cl1_contig_289	576	Penicillium citrinum ourmia-like virus 1	AYP71797.1	107/195(55%)	7e-63	100
	Calonectria pseudonaviculata	Calonectria pseudonaviculata strain CBS 139394 CpsSB_contig_1142	666	Rhizoctonia solani ourmia-like virus 1 RNA 1	ALD89131.1	90/221(41%)	3e-29	96
GSS	Symbiodinium minutum	SYMB2F fosmid Symbiodinium minutum genomic clone SYB2F-146E05	1163	Beihai narna-like virus 7	APG77081.1	120/294(41%)	1e-50	74
	marine RNA virus	JP.418D.600-24 B.C.	709	Hubei narna-like virus 8	APG77202.1	95/194(49%)	2e-44	77

community uncultured marine RNA virus	marine RNA virus community uncultured marine RNA virus genomic

Note: these similar sequences were collected by tblastn searches against the NCBI databases of non-mouse expressed sequence tags [EST] and transcriptome shotgun assembly [TSA]. Accessions with e-value less than e-5 were collected.

Genome sequence of MOLV4

```
>MOLV4
AGGGGGTACACAGGCTAGCCTGGTCCTCGGGGTTTGTGCTCCACCTGCGCATATCTCGTGGTTTAGTGCTCCTACGATTGGAGTGTGACACCGTAACAGGCAGTGCTGACGGGTCGTGGTTTCCGGGCATCACCCGGTGGAAAAGG
GGTGTTGGGGCCTTGCCTGTAATACAGGGAGAAAGGTCCTGTCCCCGAAGAGAGAAGGTAAAACGCTAATCAGGCGTGGTCCGCGACTGTGCCTCGCGCGCGGCGGATCAGACCCGGGGAGTAACTGTCCCGGGTGCGGATCTCGG
AGGGTCGGGTGGACATGGTGGTCCCCATCCCCTTATCGGCACAAAGATCGTGAAAGACCGGGTCCGGGGACCCTGACGTCTATAAAAGCGAATTGAAGTCCAACCAAAAGGAATCGAAAATATGGTGACAAATTGGCCAATTAAGT
TGTATTGGCTACCATACGGAGCTCCCTCCGTTTTCGTTCCTCTCGGCCCAGTGTCGAGTTCTTTTCTTTTAAACAAACGACTGCGTCGTCGAAGTCAGGGCGGGTGCCTGATTGCTTTCGACGTTAACCGACAATCGCCCCCTGGG
ACCGGCCCCAGGATAACCAAACCGGTTGGTCCGAACCGTGACATGGACTGTAAAAATGTTCCGCGCAATGGGAGGGTTGCGCGAGCCGCTGTGGCGCTTCTGAGCAGCGACCAGGAAATGGAGAGGGTCCGTCCCTTACCTCCACG
GATTCGTTGCGGGGATCTCCGTCCCGCAATCCGTCAATGCTTCCCGGATCAACTACCTGTTGTGAAGGAATTAAGTATTAAAACATCTCAAAAGCTAGAAATAGCACCGTGCAAGTACTGCGAGCCGGCGATGTTACATAGACTTA
ATGAGTGGCTAAAAGAGAGATACATCCCACTTGATCCGGTGGATCATGCCCACATTGCCCTTTTCAAAAGGGCCCTCGCGGTGAATGTGGACATGGGATGGAATAAAAGAAAGTACCCGTATATCCCGACGGGTCACTCGACGCTC
AACTTCCCTCGTGGAGAGGGAGGATCCTGGAATGAGGAGGGTTACAGCCCCTACTGCCGCGCCGCTACTGTCCACAGTAACGGGAAGCCCAGGATCGTCACAATGTACTCAGGACGGAACTCCGAGGTCCTGAGCCCACTCCACCA
GTCGCTGTACGCGAGTTTGAAAAGGAAGGGATGGCTTCTTGTCGGTAGCCCCACCGATGAGAGCGTAGGATGGCTAGGCAGCGGCGGCGTCTACGTATCTGTCGACTACAAGTCGGCCACAGATAACATACGTGCCGAGTACGTTC
GCGCTGCTATAGACGTCCTTAAGGCTCGGGCAGACCAGCTGACAGAAGAGGAGTCTCGTTGCCTTGATGTCGTCGGTGAACTCAGGTTTGCTGACTTCACCGAGGACACTCCCGCCACGAGGGGACAACCAATGGGTAGTTTGATG
AGCTTCCCTTTGCTTTGTTTGATAAACAAAACAGTTGTCGACCTTGCACTAGTTGATCTGGCAGAGTCGGGGAAAATCACCTGGGAGCAATTCCGGGTACATCGCTGTCTCATCAACGGCGATGACTTGCTTTATCGCGAGTTTGA
CAGCTCTCGCGATATACTTGCCGGTATCCTCCGCCATGGGACTTTGGTCGGTCTCGTGGTCAACGAGGAGAAAACGATGGTATCGGCCACCGACGCGGAGATCAACTCCACTGTGTTCGTCGGGGGTCGAAAGCAAAAGAAAACGA
ATGTGAAGGTGGTATGTTGGTCCAGTGATGTAACGGATCCCATCGGTTTTATTGCCGATTCCGTTGTCAAGACATCCTCGTTCCGCAAGCTCCTCGGGAGATGGGAAATCCCGATTAGGAAGGCCCCGCGGAAGGTCCAGGGTCCC
TTGCCTCCGTCTTTCTTTAAAGCTCTGTTTAAAGAGAAACGGATCAGGGACGCGCTGACATGGATCCCTCCTCCACCGCCAAAATCACCGAACCCCTTCCCCGTTGTAGTCAAGCCTGCAGACTACTGTTTAACTCGCGAGGAAGA
GGTGCGCTACATCTCAGAACGAGTAGCCAGGCTGAAGTCGTCGGGGTACAAGCCTCGGAAACCTGCGAGGTGCTCGACCGGCGACGGAAAAAGGTGCAATATCCAGTATGCACTAAGGAAAGAAAAACCAGCCGCAGAAGAACGGA
TACTCGCCGTTCTGGCCGACGGTTGGAAGGAGAAACAGTTTCAAAAGCTGATCGGACAATCCGACCCCTTCGTACCCCTCTCCTGGGGGTACGCAGACTGGTGCCAGGAGTGCTATGAGCATAGCCACTCCATGATCCAGTGCTTA
ACTGCGAGGATAAGGAGACAAAAGAGTGTGGGAGTAGTGCCGGACTACGGACGCAGGTCGACTGCGGTCGGGGAACCGACTCCGGTCGCGGAGGGTTTTGGCGATTACTGTTCACTTACCAGCAGTGATTAGTCTTGCCCGACGCG
CCTAGTACATGGAGC
```
